# Host–Parasite Interaction and Phylogenetic of a New Cnidarian *Myxosporean* (Endocnidozoa: Myxobolidae) Infecting a Valuative Commercialized Ornamental Fish from Pantanal Wetland Biome, Brazil

**DOI:** 10.3390/pathogens11101119

**Published:** 2022-09-29

**Authors:** Patrick D. Mathews, Omar Mertins, Anai P. P. Flores-Gonzales, Luis L. Espinoza, Julio C. Aguiar, Tiago Milanin

**Affiliations:** 1 Department of Zoology, Institute of Bioscience, University of São Paulo, São Paulo 05508-090, Brazil; 2 Laboratory of Nano Bio Materials, Department of Biophysics, Paulista Medical School, Federal University of Sao Paulo, São Paulo 04023-062, Brazil; 3 Research Institute of Peruvian Amazon (IIAP-AQUAREC), Puerto Maldonado, Madre de Dios 17000, Peru; 4 Laboratory of Biology and Molecular Genetics, Faculty of Veterinary Medicine, Universidad Nacional Mayor de San Marcos, Lima 15021, Peru; 5 Department of Basic Sciences, Faculty of Animal Science and Food Technology, University of São Paulo, Pirassununga 13635-900, Brazil

**Keywords:** cnidaria, Myxobolus, ornamental fish, Pantanal biome, Brazil

## Abstract

Myxozoans are a diverse group of parasitic cnidarians of wide distribution. A new species, *Myxobolus matogrossoensis* n. sp., is herein described infecting wild specimens of tetra mato-grosso *Hyphessobrycon eques*, caught in the Pantanal biome, the world’s largest tropical wetland area. Cysts were found in 3 of the 30 examined fishes. Mature myxospores were ovoid in shape in frontal and measured 6.6 ± 0.4 µm (6.2–7.0 µm) in length and 3.5 ± 0.2 µm (3.3–3.7 µm) in width. The two polar capsules were elongated in shape, equal in size and occupying almost half of the myxospore body. They measured 3.3 ± 0.2 µm (3.1–3.5 µm) in length and 1.8 ± 0.1 µm (1.7–1.9 µm) in width. The polar tubules presented three to four turns. Phylogenetic analysis placed the new species within a clade containing myxobolid species from South American characiforms fish and appears as a close species of *Myxobolus piraputangae* and *Myxobolus umidus*. Nevertheless, the sequences of the new species and *P. umidus* and *P. piraputangae* have a large genetic divergence of 12 and 12.2% in their 18S rDNA gene, respectively. To the best of our knowledge, this is the first report of a *Myxobolus* species parasitizing the tetra fish mato-grosso, thus increasing our knowledge of cnidarian myxosporean diversity from South America.

## 1. Introduction

Myxozoans are a biologically diverse group of microscopic cnidarians of wide distribution around the world [1]. They are parasites mostly innocuous with complex life cycles that include invertebrate and vertebrate hosts [1]. Although the majority of species have fish hosts, they have radiated sporadically into other groups of vertebrates, including amphibians, reptiles, waterfowl and small mammals [2]. Within the myxozoans, *Myxobolus* Bütschli, 1882, is one of the highly rich genera with more than 900 species described taxonomically, infecting a large variety of fishes within a wide geographical range [3,4,5].

Regarding South America, several studies have described *Myxobolus* species infecting many species of wild and farmed freshwater fish [3,6,7]. Despite this, information on ornamental freshwater fish is still scarce. The Pantanal biome is one the main biodiversity hotspots, harboring around 300 fish species [8], however, the myxozoan fauna are poorly known. Indeed, only 10 *Myxobolus* species were reported in fish from this geographic region [3,7,9]. With approximately 143 recognized species the genus *Hyphessobrycon* Durbin, 1908, is one of the largest characid genera with a wide distribution in all major watersheds of the Neotropical Region [10]. The *Hyphessobrycon* eques Steindachner, 1882, vernacularly called “tetra mato-grosso”, is originally found in the Amazon, Guaporé and Paraguay basins [11] and it is widely found in aquarium stores in several regions of Brazil. It typically inhabits ponds and small lakes, where it forms aggregations around marginal vegetation or submerged tree roots. Despite the commercial importance in the aquarium trade, information about the parasite fauna is still scarce and nothing is known about myxozoan parasites.

In the present study, we describe for the first time a histozoic myxozoan infecting the gills of the body of specimens of H. eques from the Pantanal biome, the largest freshwater wetland in the world, thus increasing our knowledge of cnidarian myxozoan diversity from South America.

## 2. Material and Methods

In July 2019, 30 specimens of H. eques (ranging from 3.8 to 4 cm in length) were examined for the presence of myxozoan parasites. The fish were acquired from local fisherfolk and were caught in the marginal vegetation beds in the oxbow lakes of Paraguay River near the municipality Porto Murtinho, Brazil. The fish were transported to the field laboratory, where they were euthanized by pit transaction and examined using a light microscope to verify the presence of lesions and myxozoans.

Morphological characterization was performed based on the criteria outlined by Lom and Arthur [12]. Measurements and photographs were taken from 30 randomly selected mature myxospores, using a computer equipped with Axiovision 4.1 image capture software coupled to an Axioplan 2 Zeiss microscope (Carl Zeiss AG, Oberkochen, Germany). The dimensions were given in micrometers (μm) and expressed as the mean ± standard deviation, followed by the range in parentheses and included spore length, thickness, polar capsule length and width. The gill plasmodial index (GPI) was determined based on the criteria established by Kaur and Katock [13] and categorization of plasmodia on the basis of size was calculated according to Kaur and Attri [14]. The plasmodia type localization was determined according to Molnár [15]. Mature myxospores released from ruptured plasmodia were air-dried, fixed with methanol, stained with Giemsa and mounted on permanent slides deposited in the cnidarian collection of the Zoology Museum at the University of São Paulo, São Paulo, Brazil.

For scanning electron microscopy, infected tissues were fixed in 2.5% glutaraldehyde prepared in 0.1 M sodium cacodylate buffer (pH 7.2), then post-fixed in 1% osmium tetroxide overnight, and dehydrated in a graded ethanol series. The samples were dried in a critical point chamber (BALZERSCPD 030, Columbia, South Carolina, SC, USA) using carbon dioxide, and were included in an aluminum stub using double-sided carbon tape and coated with a thin layer of platinum with a thickness of 20–30 nm (SPUTTERING, ©LEICA EM SCD 500, Wetzlar, Germany). Samples were visualized with a DSM 940 scanning electron microscope (Carl Zeiss, Hamburg, Germany) operating at 15 kV.

For molecular analysis, cysts were dissected from the gill lamellae and fixed in absolute ethanol. Extraction of genomic DNA was performed using QIAamp DNA Micro Kit (Qiagen, California, CA, USA). The concentration of the DNA was measured using a NanoDrop 2000 spectrophotometer (Thermo Scientific, Wilmington, North Carolina, NC, USA). Small subunit ribosomal DNA (18S rDNA) was amplified using universal eukaryotic primers ERIB1 (ACCTGGTTGATCCTGCCAG; [16]) with ERIB10 (CTTCCGCAGGTTCACCTACGG; [16]). Polymerase chain reactions were conducted in a final volume reaction of 20 μL, comprised of 1 μL of extracted DNA (10–50 ng), 0.3 μL of each primer (10 μM), and 0.625 U of Qiagen Taq DNA polymerase. Polymerase chain reactions were performed in a Thermocycler (Bio-rad T100) with initial denaturation at 94 °C for 5 min, followed by 39 cycles at 94 °C for 1 min, 58 °C for 1 min, 72 °C for 2 min and then final elongation at 72 °C for 5 min. The amplicons were subjected to electrophoresis in 2% agarose gel in a TAE buffer (Tris–Acetate EDTA: Tris 40 mM, acetic acid 20 mM, EDTA 1 mM). The size of the amplicons was estimated by comparison with the 1 Kb Plus DNA Ladder (Invitrogen by Life Technologies). Polymerase chain reactions using the original primers and two additional primers, MC5 and MC3 [17], were used in the sequencing to connect the overlapping fragments. Sequencing was performed with a BigDye^®^ Terminator v3.1 cycle sequencing kit (Applied Biosystems Inc., Valencia, CA, USA) in an ABI 3730 DNA sequencing analyzer (Applied Biosystems).

A standard nucleotide BLAST search was conducted to verify the similarity of the sequence obtained in this study with other sequences available in GenBank [18]. Phylogenetic analysis was conducted using the most closely related myxozoans sequences with similarity >80%. The sequences were aligned with ClustalW within BioEdit version 7.1.3.0 [19]. Phylogenetic analysis was performed using Maximum Likelihood method with a Kimura 2-parameter (K2P) evolution sequence model in MEGA 6.0 [20]. Bootstrap analysis with 1000 replicates was employed to assess the relative robustness of the branches in Maximum Likelihood tree. *Ceratomyxa seriolae* sequence was used as outgroup. To evaluate the genetic distance between the myxozoan species clustering together with the new obtained sequence, a pairwise method with the p-distance model in MEGA 6.0 [20] was performed.

## 3. Results

Out of 30 wild specimens of H. eques examined, three (10%) had the gill infected by a new cnidarian myxozoan species of the genus Myxobolus, described herein.

Taxonomic summary

1. Phylum: Cnidaria Verrill, 1865
2. Class: Myxosporea Bütschli, 1881
3. Order: Bivalvulida Shulman, 1959
4. Family: Myxobolidae Thélohan, 1892
5. Genus: *Myxobolus* Bütschli, 1882
6. Species: Myxobolus matogrossoensis n. sp.
7. Type host: *Hyphessobrycon eques* (Characiformes: Characidae)
8. Site of infection: Gills (Interlamellar-epithelial type, LE2)
9. Gill plasmodium index (GPI): 1 (light infection)
10. Category of plasmodium: Type A (visible under light microscope, size range 40–65 μm)
11. Type of locality: Adjacent area of lakes of Paraguay River near the municipality Porto Murtinho, Mato Grosso do Sul State, Brazil (21°41′56″ S, 57°52′58″ W).
12. Prevalence: From 30 examined fish, three were infected (10%).
13. Type of material: Hapantotype (slides with stained myxospores) were deposited in the cnidarian collection of the Zoology Museum at the University of São Paulo—MZUSP, São Paulo, Brazil (slide no. MZUSP 8695). Partial 18S rDNA sequence gene was deposited in GenBank under accession number OP244900.
14. Etymology: The specific name (*M. matogrossoensis*) is based on host species common name.

**Figure 1:**
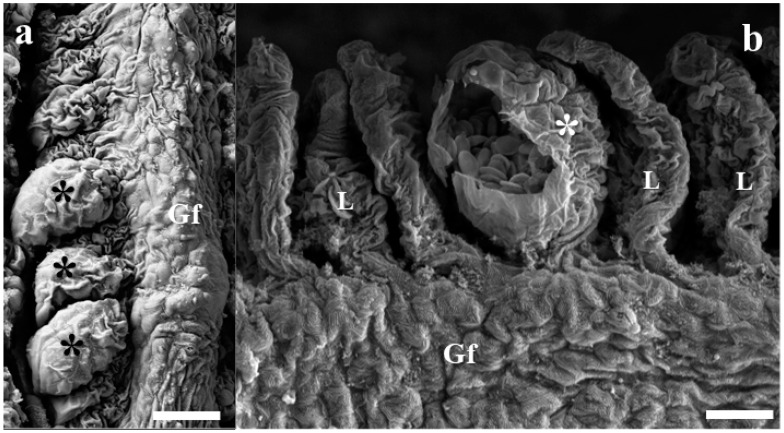
Gill lamellae infected with *M. matogrossoensis* n. sp. (**a**) Gill filament (Gf) showing three cysts (asterisks) in their lamellae (L). (**b**) Ruptured cyst (asterisk) evidencing inside large quantity of smaller myxospores. Scale bars = 20 µm.

Rounded cysts containing large quantities of smaller myxospores, measuring up to 46–55 μm, were found in the gills. Scanning electron microscopy analysis of infected tissues revealed that cyst development occurred in the lamellae (Figure 1a,b).

**Figure 2:**
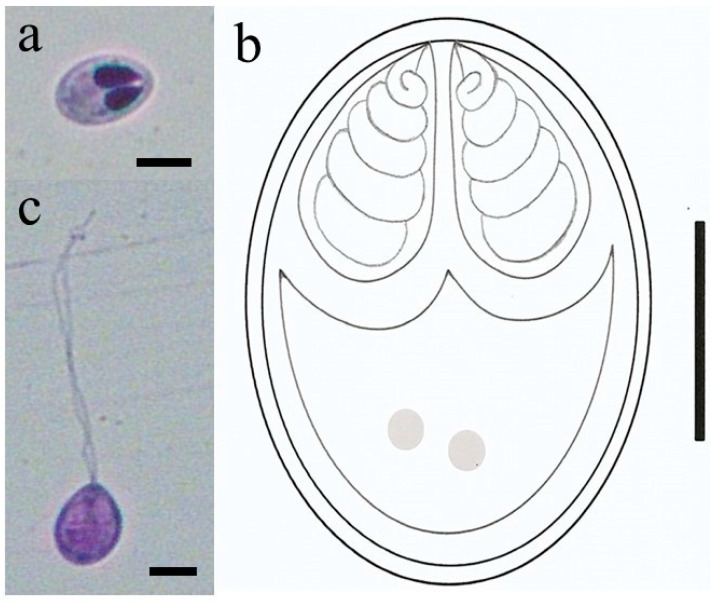
Mature myxospores of *M. matogrossoensis* n. sp. stained with Giemsa. (**a**) Mature myxospore showing two equal-size polar capsules. Scale bar = 5 μm. (**b**) Myxospore with extended polar tubule. Scale bar = 5 μm. (**c**) Schematic representation of a mature myxospore. Scale bar = 2 μm.

Mature myxospores were ovoid in shape in frontal and measured 6.6 ± 0.4 µm (6.2–7.0 µm) in length and 3.5 ± 0.2 µm (3.3–3.7 µm) in width (Figure 2a,b). The two polar capsules were elongated in shape, equal in size and occupying almost half of the myxospore body (Figure 2a,b). They measured 3.3 ± 0.2 µm (3.1–3.5 µm) in length and 1.8 ± 0.1 µm (1.7–1.9 µm) in width. The polar tubules presented three to four turns (Figure 2b). Some myxospores were observed with extended polar tubule (Figure 2c). Comparative data of *Myxobolus matogrossoensis* n. sp. with all *Myxobolus* species described in fish from Pantanal wetland biome are showed in Table 1.

**Figure 3:**
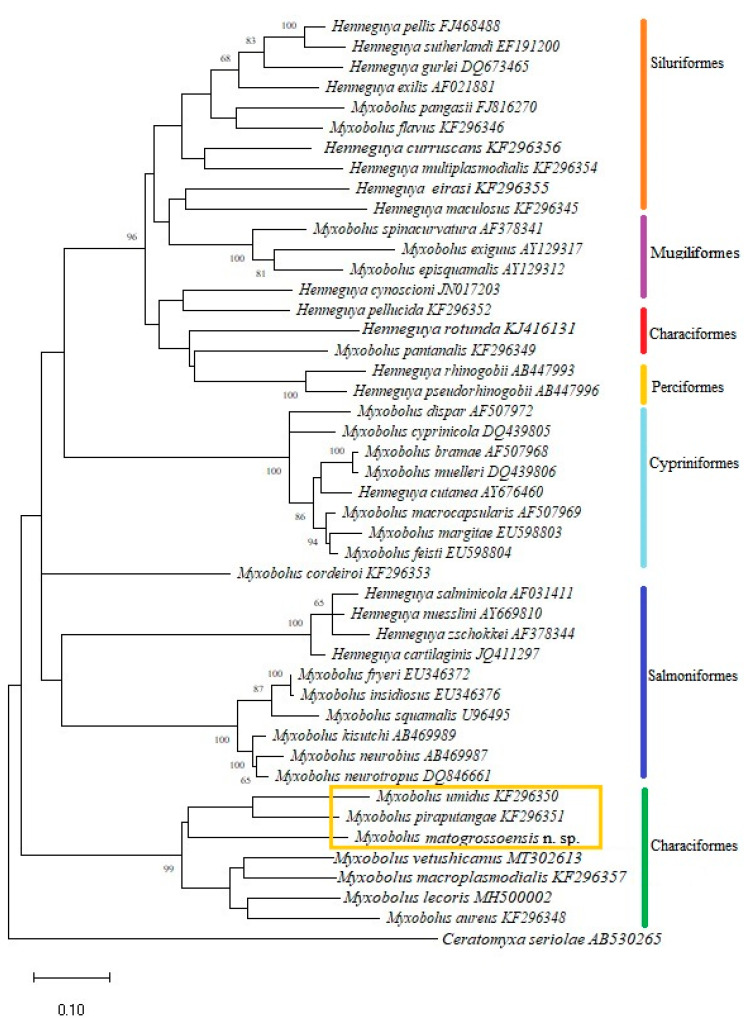
Phylogenetic tree generated based on 18S rDNA sequences of *Myxobolus* species and other closely related myxozoan species. Bootstrap values above 60 are indicated.

BLAST search performed with the sequencing of the 18S rDNA from the myxospores obtained in the study revealed that this sequence did not match any of myxozoans available in GenBank database. Sequence with the highest similarity identified by BLAST search was from *Myxobolus piraputangae* Carriero, Adriano, Silva, Ceccarelli and Maia, 2013 (query coverage 98%, maximum identities 88.3%), reported in the kidney of *Brycon hilarii*, from the Pantanal National Park, Mato Grosso State, Brazil [9]. Phylogenetic analysis inferred by the Maximum Likelihood method based on the most closely related myxozoan sequences placed the new obtained sequence together with *M. piraputangae* and *M. umidus* Carriero, Adriano, Silva, Ceccarelli and Maia, 2013 (Figure 3). Pairwise analysis of the 18S rDNA sequences of the myxosporean species that cluster in the same clade with the sequence obtained herein revealed an important genetic divergence among these species, with a difference of 12.2% to *M. piraputangae* and 12% to *M. umidus*.

## 4. Discussion

In South America the description of new myxozoan species has been increasing over the years [3], however, myxozoan diversity is still poorly known [21]. Indeed, considering that this biogeographic region harbors the greatest diversity of potential freshwater fish hosts of any continent, it is suggested that many more myxosporean parasites remain to be discovered [22]. This study expands our knowledge about South American myxozoans, with description of a new species from an important ornamental fish from Pantanal biome. To the best of our knowledge, this is the first report of a *Myxobolus* species parasitizing H. eques.

Although molecular tools have revealed great discrepancies between spore-based myxosporean taxonomy and molecular phylogenies inferred from the SSU rDNA [23], mostly myxozoan species have been described exclusively based on myxospore morphology. Thus, morphology-based comparisons remain the main criterion and are necessary to differentiate the large number of myxozoan species that lack molecular data [24]. Indeed, from South America, considering the around hundred described myxozoans, few species have their SSU rDNA sequences available in Gen-Bank [25]. Taking this issue into account, morphological comparison was performed considering species previously described as infecting fish from the Pantanal biome. The comparison between the mature myxospores of the new isolate and the others showed remarkable differences as evidenced in Table 1. According to Molnár [17] the exact location and tissue specificity is also essential for differentiation of gill-parasitic fish myxosporean species. Accordingly, differences were observed in the infected tissue between the new isolate and the other previously described Myxobolus species from the Pantanal biome.

The molecular phylogenetic analysis performed in our study showed members of genus *Myxobolus* and *Henneguya* grouped together in the produced tree (Figure 3). This is in agreement with previous studies in South America [6,9,26] and other biogeographic regions around the world that showed absence of phylogenetic separation between these two genera [4,27]. Our phylogenetic analysis also showed a strong tendency of myxobolid species to form clusters mainly based on the order and/or family of the host, despite having different geographic origins, as has been previously reported [6,9,27]. According to Carriero et al. [9] the strong tendency of *Myxobolus*/*Henneguya* species to cluster, based on host phylogenies, suggests that the origins and radiations of myxozoans are very ancient, perhaps as old as the hosts themselves that go back to an Osteichthyes Early Silurian origin and Mesozoic radiation. Our topology shows the new isolate placed in a subclade composed exclusively of *Myxobolus* spp. of bryconid fishes from South America, with *M. umidus* and *M. piraputanagae* as the closest related species (Figure 3). However, pairwise similarity analysis revealed the new isolate and these two species have a large genetic divergence of 12.2% to *M. piraputangae* and 12% to *M. umidus*, in their SSU rDNA gene sequences. Taken together, we confidently consider that these data are sufficient to define this isolate as a new freshwater *Myxobolus* species, *Myxobolus matogrossoensis* n. sp. The presence of M. matogrossoensis n. sp. inside a well-supported clade of fish from the family Bryconidae may be attributed to the few sequences available in the GenBank for many yet-to-be-discovered *Myxobolus* species from these underrepresented ornamental characids. Thus, the addition of molecular data from other taxa will enable a better understanding about the evolutionary context of *M. matogrossoensis* n. sp. Conversely, the low occurrence of *M. matogrossoensis* n. sp. in H. eques may suggest accidental infection of these hosts with infective actinospores, especially during the dry season (fish collection season in this study) when the water level is low and fishes are more concentrated. This may have favored the encounter with infective stages. We believe that this new isolate may be found in the future in a bryconid host species still not studied.

## Figures and Tables

**Table 1:** Comparative data of *Myxobolus matogrossoensis* n. sp. with all *Myxobolus* species described in fish from Pantanal wetland biome. Spore dimensions are given. SL, length of the spore; SW, width of the spore; ST, thickness of the spore; PCL, length of the polar capsules; PCW, width of the polar capsules; NC, number of coils of the polar tubules; -, no data. All measurements are range in μm and/or means ± SD.

**Myxobolus Species**	**SL**	**SW**	**ST**	**PCL**	**PCW**	**NC**	**Site of Infection**
This study	6.6 ± 0.4	3.5 ± 0.2	-	3.3 ± 0.2	1.8 ± 0.1	3–4	Gill lamelae
*M.* sp. Mathews, Mertins, Milanin, Aguiar, Gonzales-Flores, Tavares & Morandini, 2022	22.7 ± 1.2 (21.5–23.9)	12.5 ± 0.4 (12.1–12.9)	11.3 ± 0.5 (10.8–11.8)	4.6 ± 0.3 (3.9 ± 4.3)	2.9 ± 0.1 (2.8–3.0)	4–5	Mandibule
*M. cordeiroi.* Adriano, Arana, Alves, Silva, Ceccarelli, Henrique-Silva & Maia, 2009	10.8 ± 0.5	7.1 ± 0.2	5.3 ± 0.3	5.2 ± 0.3	1.4 ± 0.1	5–6	Gill arch, skin, body, eye
*M. salminus.* Adriano, Arana, Carriero, Naldoni, Ceccarelli & Maia, 2009	9.6–10.5 (10.1 ± 0.4)	5.8–6.6 (6.1 ± 0.4)	4.7–5.3 (5.0 ± 0.6)	4.3–4.8 (4.6 ± 0.2)	1.5–1.9 (1.7 ± 0.1)	7–8	Gill filament
*M. oliveirai.* Milanin, Eiras, Arana, Maia, Alves, Silva, Carriero, Ceccarelli & Adriano, 2010	11.2 ± 0.4	7.4 ± 0.5	4.6 ± 0.6	5.6 ± 0.2	2.3 ± 0.2	6–8	Gill filament
*M. brycon.* Azevedo, Casal, Marques, Silva & Matos, 2011	6.9 ± 0.6 (6.5–7.2)	3.9–4.8 (4.2 ± 0.5)	1.9–2.8 (2.5 ± 0.7)	3.8–4.7 (4.2 ± 0.6)	1.7–2.5 (1.9 ± 0.6)	8–9	Gill filament
*M. flavus.* Carriero, Adriano, Silva, Ceccarelli, Maia, 2013	9.2 ± 0.2	6.5 ± 0.3	4.2 ± 0.2	4.5 ± 0.2	1.6 ± 0.1	4–5	Gill arch
*M. pantanalis.* Carriero, Adriano, Silva, Ceccarelli, Maia, 2013	9.3 ± 0.4	6.5 ± 0.4	-	4.2 ± 0.5	2.0 ± 0.1	4	Gill filament
*M. aureus.* Carriero, Adriano, Silva, Ceccarelli, Maia, 2013	12.6 ± 0.5	8.3 ± 0.3	5.5 ± 0.3	5.7 ± 0.3	2.9 ± 0.2	7–8	Liver
*M. umidus.* Carriero, Adriano, Silva, Ceccarelli, Maia, 2013	13.5 ± 0.7	7.8 ± 0.4	7.7 ± 0.1	5.1 ± 0.4	2.7 ± 0.3	5	Spleen
*M. piraputungae.* Carriero, Adriano, Silva, Ceccarelli, Maia, 2013	10.1 ± 0.5	8.7 ± 0.5	6.7 ± 0.3	5.2 ± 0.4	3.0 ± 0.3	4–5	Kidney

## Data Availability

The data set presented in this study are available upon reasonable request to the corresponding author.
